# Trends in osteoporosis diagnosis and management in Australia

**DOI:** 10.1007/s11657-022-01139-0

**Published:** 2022-07-19

**Authors:** Leon Smith, Stephen Wilson

**Affiliations:** Department of Rehabilitation Medicine, Royal North Shore Hospital, Reserve Road, St Leonards, NSW 2065 Australia

**Keywords:** Osteoporosis, Medicare, Bone, Mineral, Density, Value-Based, Care

## Abstract

**Summary:**

Trends in bone mineral density monitoring, and drug treatment for osteoporosis, in Australia were examined. Rates of DEXA scanning have increased in response to changes to government policy affecting reimbursement. The drug denosumab is being utilised at an increasing rate, while bisphosphonate use has declined. Osteoporosis prevalence remained stable over the same timeframe, while rate of hip fractures declined, suggesting that introduction of osteoporosis screening was associated with a reduction in adverse osteoporosis outcomes, but may also have been associated with overutilisation.

**Introduction:**

Radiology interventions to diagnose and medications to manage osteoporosis in Australia are reimbursed under the Medicare benefits schedule (MBS) and Pharmaceutical Benefits Scheme (PBS). Monitoring of these databases enables changes in utilisation of these practices to be monitored over time.

**Methods:**

This study examined rates of utilisation for bone mineral density (BMD) measurement and osteoporosis pharmacotherapy subsidised under the MBS. Rates of osteoporosis and hip fracture were estimated using data reported by the Australian Bureau of Statistics (ABS) and Australian Institute for Health and Welfare (AIHW).

**Results:**

Rates of BMD measurement increased since the technology was first reimbursed, with changes to policy regarding reimbursement for screening for individuals over 70 leading to an increase in BMD measurement after 2007. Prescribing rates also increased over time, initially with the introduction of oral bisphosphonates and subsequently for denosumab, which has subsequently become the most commonly prescribed agent for osteoporosis management in Australia, while bisphosphonate use has declined. Osteoporosis prevalence in Australia has remained relatively static at 3–4% of the population since 2001 to 2017, while rates of minimal trauma hip fracture hospitalisations have declined from 195 per 100,000 to 174 per 100,000 in the same timeframe.

**Conclusion:**

Available data indicates that osteoporosis screening rates changed over time from 2001 to 2018 and that changes to government policy had a significant effect on the rates at which screening was performed. Over the same timeframe, there was a sustained reduction in hip fracture hospitalisation rates, with no change to reported osteoporosis prevalence. This suggests that policy changes permitting unlimited access to BMD measurement were associated with a reduction in osteoporotic fractures, but may also have been associated with overutilisation. Prospective studies to assess the efficacy of specific policies to ensure screening is performed in accordance with best-practice guidelines may be desirable.

**Supplementary Information:**

The online version contains supplementary material available at 10.1007/s11657-022-01139-0.

## Background

Osteoporosis is a health condition affecting approximately 4% of the Australian population [1]. It is characterised by reduced BMD, which can predispose to an increased risk of minimal-trauma fractures [2]. Osteoporosis management involves advice around appropriate dietary intake of calcium, replenishment of vitamin D and weight bearing exercise[3]; falls prevention is also of importance in reducing the risk of fractures in those with osteoporosis [3]. Additionally, pharmacotherapy can be prescribed to increase bone mineral density and reduce the risk of future fractures [2, 3].

Historically, osteoporosis was diagnosed in patients who had sustained a clinically compatible minimal trauma fracture [4]. However, technologies introduced in the late 1980s such as dual-energy x-ray absorptiometry (DEXA) permitted objective measurement of BMD before a fracture had occurred. DEXA results are usually reported in terms of bone mineral density, the *T*-score (standard deviations above or below the young healthy mean) and *Z* score (standard deviations above or below the mean BMD for the same gender and age). In 1995, the WHO adopted a “T-score” of − 2.5 (meaning a BMD of 2.5 standard deviations below the young healthy mean) as the definition of osteoporosis [4, 5]. It is possible to use DEXA scanning to screen for osteoporosis in asymptomatic individuals. This is recommended in certain guidelines, including those published by the US Preventative Services Task Force (which recommends screening in asymptomatic women over 65 years of age and those under 65 with increased risk) [6]. In Australia, BMD measurement (specifically *T*-score) is used for public reimbursement of osteoporosis pharmacotherapy [7] through the Australian Government’s Pharmaceutical Benefits Scheme (PBS).

BMD measurement for osteoporosis is reimbursed in Australia under the MBS for diagnosis and management of established osteoporosis, either after a fracture or confirmed on previous BMD measurement. BMD measurement is reimbursed up to once every 2 years or once every 12 months after a significant change in therapy. Other BMD item numbers also permit BMD measurement for diagnosing and monitoring bone loss associated with specified predisposing medical conditions. In addition, MBS item 12,323, introduced in 2006, permitted unlimited access to DEXA scanning for those over the age of 70, with no clinical or time-based criteria attached. In 2017, changes to the MBS were introduced to reduce the rate of unnecessarily frequent BMD measurement [8]. The effect of this change is that individuals over 70 are entitled to a single initial screening BMD scan and then repeat scans every 2 years, for individuals with a *T*-score less than − 1.5, or every 5 years otherwise.

Specific osteoporosis pharmacotherapy is reimbursed in Australia under the PBS. This began with the bisphosphonate alendronate, introduced in 1995 [9]. Bisphosphonates are anti-resorptive agents for osteoporosis treatment, including the oral agents’ risedronate and alendronate and also the intravenous agent zoledronic acid. The latter is a more potent bisphosphonate given as an annual infusion [10]. Also available in Australia is the anti-RANKL antibody denosumab, given as a six monthly subcutaneous injection [11]. It has a slightly more favourable side effect profile than most bisphosphonates, with the disadvantage that its anti-fracture protective effects wear off after it is ceased [11]. More recent agents include the parathyroid hormone analogue teriparatide, and the anti-sclerostin antibody romosozumab [12, 12]. Both agents are currently reimbursed only for severe established osteoporosis refractory to other treatments.

The purpose of this study was to evaluate the effect of changes to government policy regarding reimbursement of BMD measurement (particularly for osteoporosis screening) and pharmacotherapy for osteoporosis in Australia, to determine if this had a significant association with clinical practice and/or outcomes in patients with osteoporosis (Tables 1 and 2).

## Methodology

To identify trends in BMD measurement utilisation, MBS items pertaining to BMD measurement with DEXA and quantitative CT were utilised. The relevant items utilised are available in Table 3. From these items, it is possible to determine the clinical indication for the DEXA scan reimbursed under that item. These were categorised into the following:

- Diagnosis and management of known osteoporosis (confirmed on BMD measurement) or in the context of a fracture (MBS items 12306, 12309 and 12321).
- Diagnosis and management of osteoporosis associated with an approved secondary medical indication (MBS items 12312, 12315 and 12318).
- Osteoporosis screening in those over 70 (MBS items 12320, 12322, 12323).

Each DEXA scan performed can only be billed under a single MBS item, and the item descriptors stipulate that the most relevant item be billed for any given scan (e.g. a scan performed for monitoring of bone loss associated with an approved condition should be billed under the relevant item for that condition, not for screening in an individual over 70). As such, by monitoring the rates at which each set of items are billed, it is possible to estimate the indications for which BMD measurement in Australia is generally performed.

To obtain data on treatment on treatment for osteoporosis, the relevant PBS codes were obtained for agents used for osteoporosis treatment. PBS codes for the following agents were obtained:

- Oral bisphosphonates (including oral bisphosphonates in combination with cholecalciferol)
- Zoledronic acid
- Denosumab
- Teriparatide
- Romosozumab
- Raloxifene
- Strontium ranelate (PBS item discontinued)

Most of these agents are reimbursed under PBS “authority” codes which specify the clinical indication for which they will be reimbursed. Only those related to osteoporosis were included. Calcitriol was not included as its PBS code included multiple indications, not all of them related to osteoporosis.

In order to obtain data for MBS items as a proportion of population, the raw number of services for each financial year was divided by the Australian population by the middle of that year (i.e. the end of the first corresponding calendar year), as provided from the Australian Bureau of Statistics ABS.Stat program [13]. The MBS permits searches back to 1994 or from the date of introduction for a given item, whichever is later. Data was standardised to the reference population of Australia on 30 June 2001 according to the ABS.Stat program [13], using the direct age standardisation method. Tests for correlation between utilisation and year were performed using Spearman’s test.

In the case of the PBS, publicly available data is not available for age or gender, and so the PBS prescriptions were reported as per-capita rates (scripts per 100,000) for the middle of the corresponding financial year without age standardisation. PBS items were also standardised for the expected number of times that a given code would be reimbursed for 12 months of treatment. For instance, codes for once-daily medications permit up to 28 tablets to be dispensed at a time, so it would be expected that, for 12 months of treatment, this would be reimbursed approximately 13 times. Denosumab, meanwhile, is administered twice a year so it would be expected that this item would be reimbursed twice a year. The items analysed, along with the expected number of times each item would be reimbursed, are included in Table 4. Items reimbursed under both the PBS and “repatriation PBS” (RPBS), which functions similarly to the PBS but reimburses for war veterans were included. For agents of interest (notably denosumab), the proportion of items reimbursed for these agents, adjusted for the number of expected items per year were compared for different years using the chi-squared test for the difference in proportions.

To obtain prevalence data for osteoporosis in Australia, the 2017/2018 Australian Bureau of Statistics National Health Survey data were analysed. The National Health Survey is conducted every 3 years and collects self-reported diagnoses from approximately 21,300 survey participants [14]. Hip fracture prevalence was obtained using publicly available data from the Australian Institute for Health and Welfare (AIHW). The AIHW analysed the rate of hip fracture hospitalisations up to 2015/2016[15] and published updated figures for 2017/2018 [1]. The analysis up to 2015/2016 produced direct age standardised to the Australian population as of 30 June 2001. For 2017/2018, raw figures were supplied that were direct age standardised to the same population. Tests for correlation between osteoporosis prevalence and time were performed using Spearman’s test.

Excel version 2016 (Microsoft, Redmond, WA, USA) was used for analysis. Jamovi v 2.2.2.5.0 (The Jamovi Project, 2021) was used to perform the chi-squared test and Spearman’s correlation analysis.

As this study involved only the analysis of aggregated deidentified data, all of which was freely available in the public domain, ethics approval was not required.

## Results

### BMD measurement

**Fig. 1:**
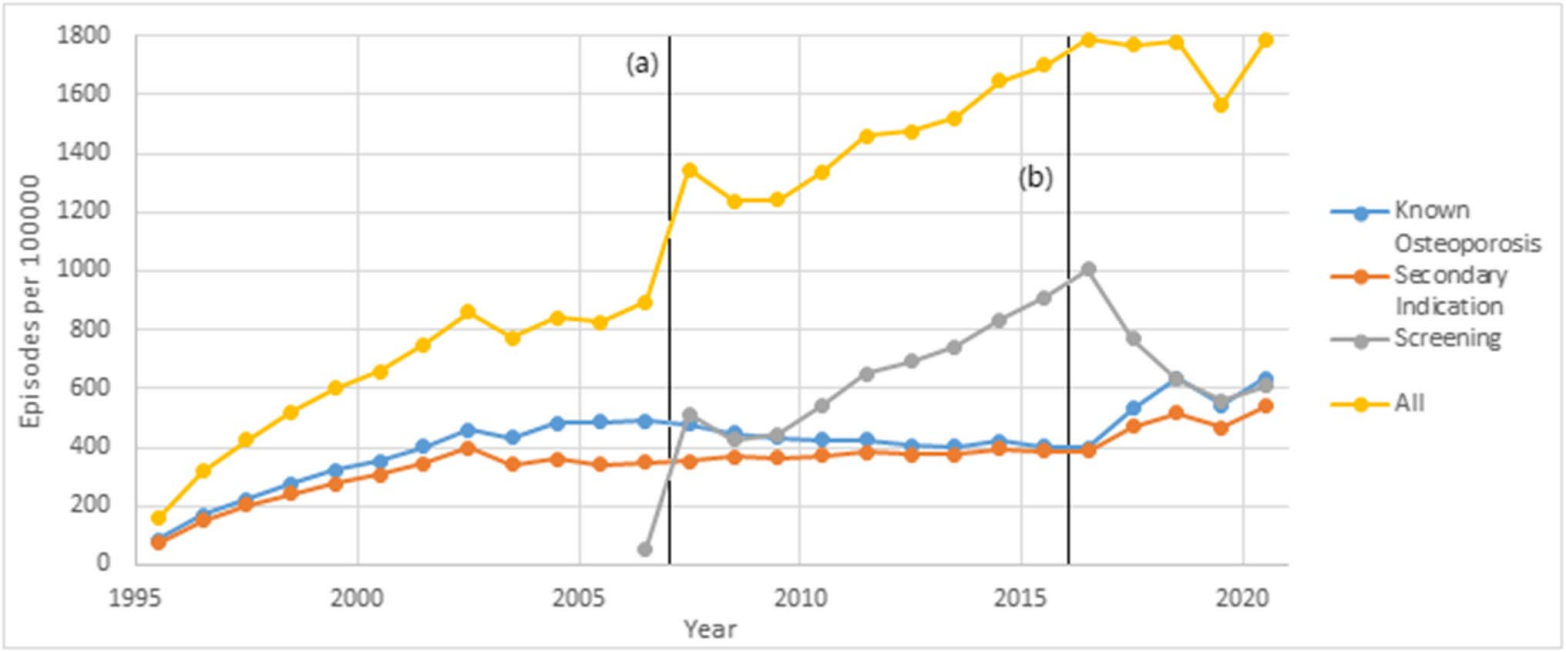
Age-standardised BMD measurement rates in Australia, per 100,000 population. Black vertical lines represent the introduction (**a**) and removal (**b**) of screening DEXA item 12,323

Age-standardised rates of BMD measurement, measured as episodes per 100,000 population, are displayed in Fig. 1.

The overall utilisation of BMD measurement has increased steadily since it was first listed on the MBS in 1995 to 2021 (Spearman’s rho 0.976, *P* < 0.001). Within these trends, however, it can be appreciated that the rate of utilisation appeared to stabilise after 2002, lasting until 2006.

A considerable increase in BMD measurement has since occurred since 2007 which began when unlimited access to screening for over 1970s was introduced under MBS item 12,323. BMD measurement for those with known or presumptive osteoporosis, or those with a secondary indication, declined slightly after MBS item 12,323 was introduced, confirming that much of this increased utilisation was driven by a trend toward screening for osteoporosis in over 1970s, rather than an increase in predisposing conditions or confirmed fractures. BMD measurement has been utilised for known osteoporosis and for secondary indications at roughly similar rates since both measures were introduced.

Screening rates peaked in 2017 at a rate of 1004 scans per 100,000 population. After this, changes to the MBS have led to a decline in screening rates to 609 scans per 100,000 population, similar to the rates of utilisation for known osteoporosis and secondary indications.

## Osteoporosis pharmacotherapy

**Fig. 2:**
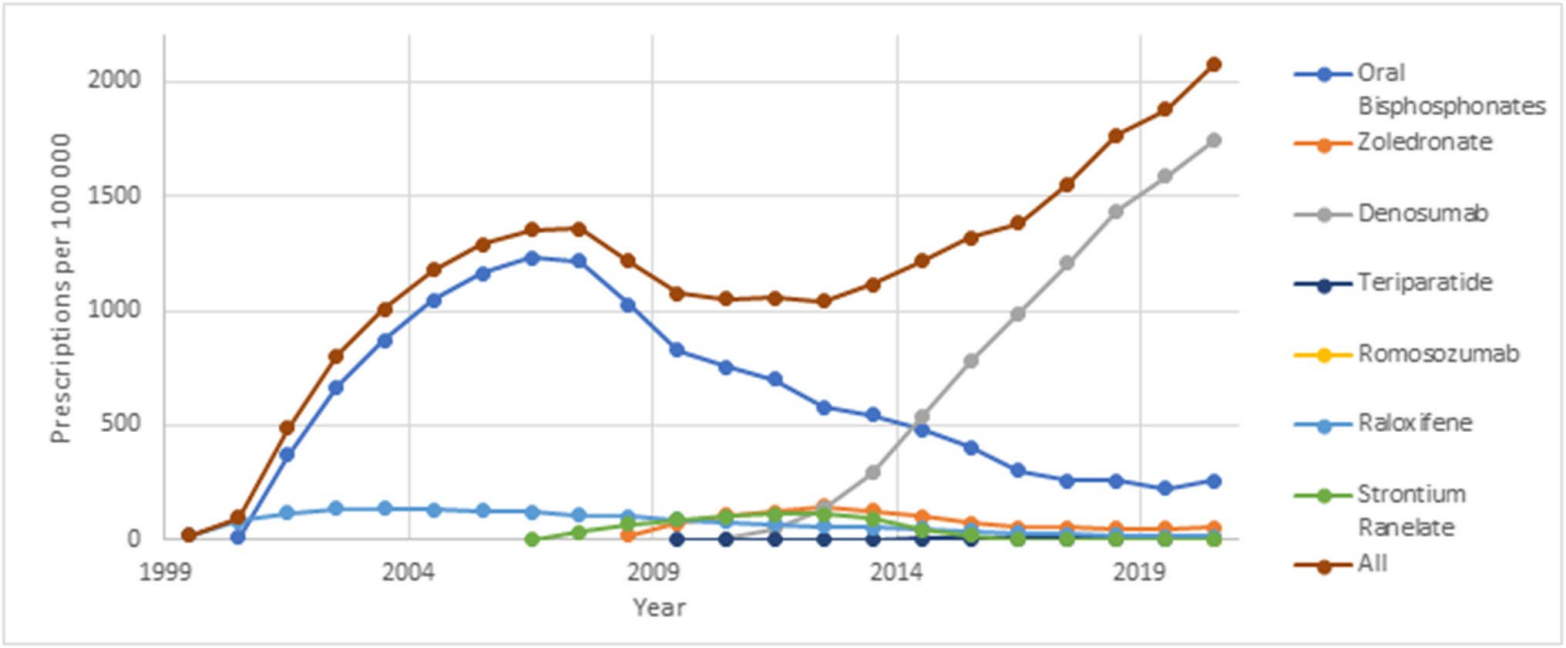
Rates of osteoporosis pharmacotherapy dispensing (not age standardised) in Australia, per 100,000 population

Rates of osteoporosis pharmacotherapy dispensation, measured as episodes per 100,000 population, are displayed in Fig. 2.

Oral bisphosphonates were the first osteoporosis pharmacotherapy made available under the PBS and were the predominant agent used for osteoporosis treatment. Bisphosphonate prescribing did however decline considerably after 2007, as did total rates of treatment. Since then, the use of oral bisphosphonates has continued to decline, while rates of overall treatment have increased again, driven by other agents. Of these, the most notable is denosumab, whose use has continued to increase consistently since introduction. It was most recently used by 1741 per 100,000 individuals in 2020/2021. When first introduced in 2010/2011, denosumab comprised 0.4% of all items reimbursed, after adjusting for expected reimbursements per year. In 2020/2021, this had increased 82.3% of all items reimbursed, representing a statistically significant increase in proportion of 0.82 (*p* < 0.001).

Rates of utilisation for other parenteral agents have remained relatively low, as have utilisation of raloxifene and strontium (which has subsequently been discontinued due to concern relating to cardiovascular side effects). Utilisation of osteoporosis pharmacotherapy overall peaked in 2007/2008 at 1359 per 100,000, before declining and then increasing again to 2072 per 100,000 in 2020/2021.

## Osteoporosis and hip fracture prevalence

Osteoporosis prevalence is estimated in Australia via the Australian Bureau of Statistics National Health Survey [14]. The most recent survey was for financial year 2017/2018, with data being available from 2001. Data was available in terms of percentages of different age groups that reported having previously received a diagnosis of osteoporosis from a health practitioner. This was standardised to the Australian population in June 2001. The results of this analysis are described in Table 1.

**Table 1:** Age-standardised rate of reported osteoporosis in Australia (source: Australian Bureau of Statistics National Health Survey)

	Female (%)	Male (%)	Total (%)
2001	2.71	0.56	1.65
2004/2005	5.01	0.87	2.96
2007/2008	5.37	1.14	3.27
2011/2012	4.40	1.17	2.79
2014/2015	5.08	1.22	3.17
2017/2018	6.2	1.5	3.8

This data would suggest an increase in osteoporosis prevalence since 2001, which has remained relatively stable for much of that time. The proportions in 2017/2018 are marginally higher than preceding years, although it remains as yet unclear if this represents a sustained increase in osteoporosis rates in recent years. No statistically significant association between year and osteoporosis prevalence could be identified (Spearman’s Rho 0.800, *p* = 0.136). Similar results are observed for the population aged over 75 (Table 2).

**Table 2:** Osteoporosis prevalence in those aged 75 or above (source: Australian Bureau of Statistics National Health Survey)

	Female (%)	Male (%)
2001	15.1	3.2
2004/2005	26.2	4.7
2007/2008	31.1	8.6
2011/2012	29.0	7.8
2014/2015	25.8	7.2
2017/2018	29.0	10.3

As no statistically significant trend in reported osteoporosis prevalence over time is apparent, it is likely that changes to screening rates have not had a significant impact of osteoporosis prevalence since at least 2001.

**Fig. 3:**
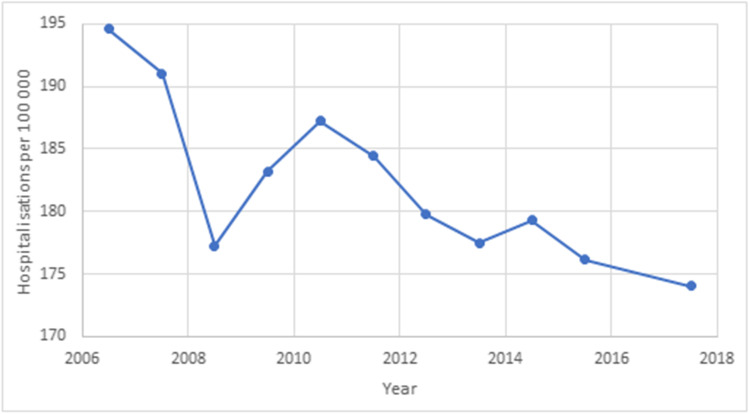
Incidence of minimal trauma hip fracture hospitalisations, reported by the AIHW, per 100,000 population

The rate of minimal trauma hip fracture hospitalisations reported by the AIHW is described in Fig. 3.

A sustained decline in hospitalisations from minimal trauma hip fractures is described, from 195 per 100,000 in 2006/2007, to 174 per 100,000 in 2017/2018. Non-parametric regression analysis reveals a significant negative correlation between date and hospitalisation rates (Spearman’s Rho − 0.773, *p* = 0.008). This appears to be a sustained reduction since 2006, after which population screening for osteoporosis was permitted under the MBS.

## Discussion

The data indicates that BMD measurement for osteoporosis demonstrated a considerable increase in utilisation since the technology was first reimbursed on the MBS in 1995, with increases since 2007 primarily driven by screening of those over 70, without other indications attached. Data for osteoporosis and hip fracture prevalence are not available to the same level of detail, although the existing publicly available data from the National Health Survey does support a non-significant increase in osteoporosis prevalence, particularly after 2001. This may be attributable to more widespread use of BMD measurement diagnosing more cases of osteoporosis. It may be the case that patients who have had a BMD scan are more likely to recall a specific diagnosis of osteoporosis compared to those with a clinical diagnosis of osteoporosis based on a fracture. The rate of hip fracture hospitalisation, meanwhile, shows a sustained downward trend since 2006, when data first became available.

Changes to government policy in 2017 have reduced the rate at which this screening is performed. This may indicate a reduction in overutilisation, in that screening is still permitted under the new MBS items, albeit at a more restricted frequency. Given that DEXA scanning at a frequency beyond once every 2 years is generally not clinically indicated [16], these changes appear to have been effective at reducing unnecessarily frequent utilisation and likely brought screening practices in line with best-practice recommendations.

It is not possible to definitively determine from the data if the decline in hip fractures can be attributable to osteoporosis screening and management, as other interventions including falls prevention, medication management and other interventions could also have contributed to this. Data for 2020/2021 is yet to be reported, although this may be instructive in determining if reduction in screening frequency after 2017 had any adverse impact on hip fracture rates. In the case of osteoporosis pharmacotherapy, prescription rates again increased although stabilised before declining after 2006/2007. The reasons for this are unclear as BMD measurement continued to increase during this time. It is possible that it may have been driven by recommendation for bisphosphonate treatment to be time limited for a period of years (usually 3–6 years), before a “drug holiday” is recommended. If this were the case, some patients would stop treatment after a certain timeframe and may not need to reinstate their treatment. Part of the decline also seems to relate to the availability of alternative options such as zoledronic acid or strontium becoming available at around the time of the decline. Finally, it is possible that some degree of time delay exists between the introduction of screening and the detection of clinical osteoporosis in screened individuals, before prescribing increases.

After 2012, rates of prescribing increase considerably, the majority of which is of denosumab, which based on the available data is now the dominant pharmacological option for osteoporosis treatment in Australia. Denosumab has a favourable efficacy profile to oral bisphosphonates and is administered in a relatively convenient 6-monthly subcutaneous injection, which likely contribute to its increasing popularity for osteoporosis treatment [11]. Other agents including zoledronic acid, teriparatide and romosozumab are not utilised to large extents. Zoledronic acid is given by intravenous infusion which may be less convenient to organise, while teriparatide and romosozumab are given by regular subcutaneous injection and are only reimbursed for osteoporosis refractory to other treatments. Denosumab has proven a popular agent for osteoporosis treatment worldwide, with similar studies in the USA reporting its use increased at a faster rate than any other agent for osteoporosis, although it did not displace alendronate as the dominant agent for osteoporosis [17]. Although denosumab has established efficacy for fracture prevention in osteoporosis, some challenges exist with its use, particularly in terms of length of treatment and the possibility of a “rebound effect” in which BMD declines if it is ceased, which may have implications for osteoporosis treatment in the future [18].

When comparing the results of our study to equivalent trends in other countries, it can be observed that the effects of similar policy changes have also had effects on clinical outcomes [19–21]. In the USA, changes to reimbursement for BMD measurement were also introduced in 2007, which had the effect of reducing the number of DEXA scans performed [20, 21]. Dhital et al. concluded that these changes may have contributed to a decrease in osteoporosis rates, but an increase in osteoporotic fragility fractures in the years following that policy change. This would lend further support to the conclusion that changes to screening policy can have a practical impact on osteoporosis and fracture rates and highlight the importance of policies that facilitate effective screening.

There remains some controversy regarding best practice for osteoporosis screening, with different authorities publishing conflicting guidelines. For instance, while the US Preventive Services Task Force recommends screening for osteoporosis in postmenopausal women [6], the UK National Screening Committee currently recommends against screening(22).

The results of this study suggest that funding for population screening in Australia appears to have been effective, although the initial policy of introducing unlimited access to screening without time-based requirements may have facilitated unnecessarily frequent screening. As such, further evaluation to determine the optimum frequency at which screening should be reimbursed, to enable an appropriate balance between effective screening and fracture prevention, compared to overutilisation, is advisable.

Advantages of this study’s approach are that the use of publicly available, routinely collected data allow rapid and low-cost monitoring of osteoporosis diagnosis and management approaches that can be regularly repeated as new data becomes available. The use of a single national healthcare system to reimburse medical imaging and pharmaceutical subsidies also enables systematic data across the entire population to be collected. Limitations do include the fact that the MBS items for DEXA scanning do incorporate multiple indications (e.g. fractures and previous low BMD) into a single item, limiting the accuracy at which some indications can be reliably tracked. This study also relies on accurate identification of MBS items by the referring clinician at a time BMD measurements are requested, which is a potential source of error. A further limitation of this study is that the available data does not provide any insight into the extent to which systematic fracture prediction tools such as FRAX are utilised. This tool is freely available online, and its use is not systematically recorded in any easily accessible format. The use of this tool and its effect on practice could be explored in future qualitative studies involving clinicians who treat osteoporosis.

## Conclusion

Data pertaining to publicly funded osteoporosis diagnosis and management in Australia since 1995 is presented. These data indicate increased rates of BMD measurement and pharmacotherapy for osteoporosis (particularly denosumab). Changes to government policy regarding reimbursement for DEXA scanning have had a noticeable impact on BMD measurement utilisation, which was associated with a reduction in hospitalisations for hip fracture during the same timeframe. This suggests that introduction of publicly funded screening for osteoporosis was associated with a reduction in hip fracture rates, although this should be assessed further in prospective studies. Similarly, the impact of policies regarding the frequency at which screening is reimbursed should be further assessed in terms of impact on fracture rates.

### Electronic supplementary material

Below is the link to the electronic supplementary material.

Supplementary file1 (XLSX 17 KB)

## Appendix 1 MBS and PBS items included in this study

**Table 3:** MBS items for BMD measurement analysed in this study

Item number	Description	Date introduced
12306	Bone densitometry (performed by a specialist or consultant physician where the patient is referred by another medical practitioner), using dual-energy X-ray absorptiometry, for: • The confirmation of a presumptive diagnosis of low bone mineral density made on the basis of 1 or more fractures occurring after minimal trauma; or • For the monitoring of low bone mineral density proven by bone densitometry at least 12 months previously Measurement of 2 or more sites—1 service only in a period of 24 months—including interpretation and report; not being a service associated with a service to which item 12,309, 12,312, 12,315, 12,318 or 12,321 applies (ministerial determination)	1995
12309	Bone densitometry (performed by a specialist or consultant physician where the patient is referred by another medical practitioner), using quantitative computerised tomography, for the confirmation of a presumptive diagnosis of low bone mineral density made on the basis of 1 or more fractures occurring after minimal trauma; or for the monitoring of low bone mineral density proven by bone densitometry at least 12 months previously Measurement of 2 or more sites—1 service only in a period of 24 months—including interpretation and report; not being a service associated with a service to which item 12,306, 12,312, 12,315, 12,318 or 12,321 applies (ministerial determination)	1995 Discontinued 2017
12312	Bone densitometry (performed by a specialist or consultant physician where the patient is referred by another medical practitioner), using dual-energy X-ray absorptiometry, for the diagnosis and monitoring of bone loss associated with 1 or more of the following conditions: • Prolonged glucocorticoid therapy; • Conditions associated with excess glucocorticoid secretion; • Male hypogonadism; or • Female hypogonadism lasting more than 6 months before the age of 45 Where the bone density measurement will contribute to the management of a patient with any of the above conditions—measurement of 2 or more sites—1 service only in a period of 12 consecutive months—including interpretation and report; not being a service associated with a service to which item 12,306, 12,309, 12,315, 12,318 or 12,321 applies (ministerial determination)	1995
12315	Bone densitometry (performed by a specialist or consultant physician where the patient is referred by another medical practitioner), using dual-energy X-ray absorptiometry, for the diagnosis and monitoring of bone loss associated with 1 or more of the following conditions: • Primary hyperparathyroidism; • Chronic liver disease; • Chronic renal disease; • Proven malabsorptive disorders; • Rheumatoid arthritis; or • Conditions associated with thyroxine excess Where the bone density measurement will contribute to the management of a patient with any of the above conditions—measurement of 2 or more sites—1 service only in a period of 24 consecutive months—including interpretation and report; not being a service associated with a service to which items 12,306, 12,309, 12,312, 12,318 or 12,321 applies (ministerial determination)	1995
12318	Bone densitometry (performed by a specialist or consultant physician where the patient is referred by another medical practitioner), using quantitative computerised tomography, for the diagnosis and monitoring of bone loss associated with 1 or more of the following conditions: • Prolonged glucocorticoid therapy; • Conditions associated with excess glucocorticoid secretion; • Male hypogonadism; • Female hypogonadism lasting more than 6 months before the age of 45; • Primary hyperparathyroidism; • Chronic liver disease; • Chronic renal disease; • Proven malabsorptive disorders; • Rheumatoid arthritis; or • Conditions associated with thyroxine excess Where the bone density measurement will contribute to the management of a patient with any of the above conditions—measurement of 2 or more sites—1 service only in a period of 24 consecutive months—including interpretation and report; not being a service associated with a service to which item 12,306, 12,309, 12,312, 12,315 or 12,321 applies (ministerial determination)	1995 Discontinued 2017
12320	Bone densitometry, using dual-energy X-ray absorptiometry or quantitative computed tomography, involving the measurement of 2 or more sites (including interpretation and reporting) for measurement of bone mineral density, if (a) the patient is 70 years of age or over and (b) either (i) the patient has not previously had bone densitometry or(ii) the *t*-score for the patient’s bone mineral density is − 1.5 or more; other than a service associated with a service to which item 12,306, 12,312, 12,315, 12,321 or 12,322 applies For any particular patient, once only in a 5 year period	2017
12321	Bone densitometry, using dual-energy X-ray absorptiometry, involving the measurement of 2 or more sites at least 12 months after a significant change in therapy (including interpretation and reporting), for (a) established low bone mineral density or (b) confirming a presumptive diagnosis of low bone mineral density made on the basis of one or more fractures occurring after minimal trauma; other than a service associated with a service to which item 12,306, 12,312 or 12,315 applies For any particular patient, once only in a 12 month period	1995
12322	Bone densitometry, using dual-energy X-ray absorptiometry or quantitative computed tomography, involving the measurement of 2 or more sites (including interpretation and reporting) for measurement of bone mineral density, if (a) the patient is 70 years of age or over and (b) the *t*-score for the patient’s bone mineral density is less than − 1.5 but more than − 2.5; other than a service associated with a service to which item 12,306, 12,312, 12,315, 12,320 or 12,321 applies For any particular patient, once only in a 2 year period	2017
12323	Bone densitometry (performed by a specialist or consultant physician where the patient is referred by another medical practitioner), using dual-energy X-ray absorptiometry or quantitative computerised tomography, for the measurement of bone mineral density, for a person aged 70 years or over Measurement of 2 or more sites—including interpretation and report; not being a service associated with a service to which item 12,306, 12,309, 12,312, 12,315, 12,318 or 12,321 applies (ministerial determination)	2006 Discontinued 2017

**Table 4:** PBS codes for osteoporosis pharmacotherapy analysed in this study. “Expected frequency” refers to the frequency at which the item would ordinarily be reimbursed for one years’ treatment

Item code	Description	Expected frequency
3036T	Strontium ranelate 2 g granules, 28 × 2 g sachets	Thirteen times per year (4-week treatment)
5457F	Denosumab 60 mg/mL injection, 1-mL syringe, 1	Twice per year (six monthly injection)
8363E	Raloxifene hydrochloride 60 mg tablet, 28	Thirteen times per year (4-week treatment)
8481J	Risedronate sodium 5 mg tablet, 28	Thirteen times per year (4-week treatment
8511Y	Alendronate 70 mg tablet, 4	Thirteen times per year (once weekly tablet)
8621R	Risedronate sodium 35 mg tablet, 4	Thirteen times per year (once weekly tablet)
8899J	Risedronate sodium 35 mg tablet [4] (&) calcium (as carbonate) 500 mg tablet [24], 28	Thirteen times per year (4-week treatment)
8972F	Risedronate sodium 35 mg enteric tablet, 4	Thirteen times per year (once weekly tablet)
9012H	Alendronate 70 mg + colecalciferol 70 µg (2800 units) tablet, 4	Thirteen times per year (once weekly tablet)
9183H	Alendronate 70 mg + colecalciferol 140 µg (5600 units) tablet, 4	Thirteen times per year (once weekly tablet)
9288W	Zoledronic acid 5 mg/100 mL injection, 100 mL vial	Once per year (annual infusion)
9351E	Alendronate 70 mg + colecalciferol 140 µg tablet [4] (&) calcium (as carbonate) 500 mg tablet [48], 1 pack	Thirteen times per year (4-week treatment)
9391G	Risedronate sodium 150 mg tablet, 1	Twelve times per year (once monthly tablet)
12301K	Romosozumab 105 mg/1.17 mL injection, 2 × 1.17 mL syringes	Twelve times per year (once monthly injection)
9411H	Teriparatide 250 µg/mL injection, 2.4 mL pen device	Twelve times per year (for 20 µg per injection, 30-day supply per item)
